# The coronal alignment differs between two‐dimensional weight‐bearing and three‐dimensional nonweight bearing planning in total knee arthroplasty

**DOI:** 10.1002/jeo2.12007

**Published:** 2024-02-23

**Authors:** Patrick Pflüger, Sandro Hodel, Stefan M. Zimmermann, Svenja Knechtle, Lazaros Vlachopoulos, Sandro F. Fucentese

**Affiliations:** ^1^ Department of Orthopedics, Balgrist University Hospital University of Zurich Zurich Switzerland

**Keywords:** computer‐assisted surgery, knee osteoarthritis, lower extremity, tomography, total knee arthroplasty

## Abstract

**Purpose:**

The goal of this study is (1) to assess differences between two‐dimensional (2D) weight‐bearing (WB) and three‐dimensional (3D) nonweight‐bearing (NWB) planning in total knee arthroplasty (TKA) and (2) to identify factors that influence intermodal differences.

**Methods:**

Retrospective single‐centre analysis of patients planned for a TKA with patient‐specific instruments (PSI). Preoperative WB long‐leg radiographs and NWB computed tomography were analysed and following radiographic parameters included: hip–knee–ankle angle (HKA) (+varus/−valgus), joint line convergence angle (JLCA), femorotibial subluxation and bony defect classified according to Anderson. Preoperative range of motion was also considered as possible covariate. Demographic factors included age, sex, and body mass index.

**Results:**

A total of 352 knees of 323 patients (66% females) with a mean age of 66 ± 9.7 years were analysed. The HKA differed significantly between 2D and 3D planning modalities; varus knees (*n* = 231): 9.9° ± 5.1° vs. 6.7° ± 4°, *p* < 0.001; valgus knees (*n* = 121): −8.2° ± 6° vs. −5.5° ± 4.4°, *p* < 0.001. In varus knees, HKA (*β* = 0.38; *p* < 0.0001) and JLCA (*β* = 0.14; *p* = 0.03) were associated with increasing difference between 2D/3D HKA. For valgus knees, HKA (*β* = −0.6; *p* < 0.0001), JLCA (*β* = −0.3; *p* = 0.0001) and lateral distal femoral angle (*β* = −0.28; *p* = 0.03) showed a significant influence on the mean absolute difference.

**Conclusion:**

The coronal alignment in preoperative 3D model for PSI‐TKA significantly differed from 2D WB state and the difference between modalities correlated with the extent of varus/valgus deformity. In the vast majority of cases, the 3D NWB approach significantly underestimated the preoperative deformity, which needs to be considered to achieve the planned correction when using PSI in TKA.

**Level of Evidence:**

Level III.

**Abbreviations**
AP: anteroposterior
BMI: body mass index
CT: computed tomography
HKA: hip–knee–ankle angle
IQR: interquartile range
JLCA: joint line convergence angle
LLR: full‐leg standing radiograph
mLDFA: mechanical lateral distal femoral angle
mMPTA: mechanical medial proximal tibia angle
MRI: magnetic resonance image
NWB: nonweight bearing
OR: odds ratio
PSI: patient‐specific instruments
ROM: range of motion
SD: standard deviation
TF: tibiofemoral subluxation
TKA: total knee arthroplasty
WB: weight‐bearing

## INTRODUCTION

Preoperative planning in primary total knee arthroplasty (TKA) includes standard radiographic evaluation with a weight‐bearing (WB) anteroposterior, a lateral view of the knee, and a patellofemoral joint view. To assess lower limb alignment, a full‐leg standing radiograph (LLR) is commonly performed [1, 2, 3].

In order to improve patient outcomes, patient‐specific instruments (PSI) and cutting blocks for TKA were introduced. PSI‐TKA should significantly contribute to a consistent alignment, avoiding intramedullary instrumentation and simplifying operating room procedures. A preoperative computed tomography (CT) scan or magnetic resonance image (MRI) is required, depending on the manufacturers specification [4]. Meta‐analyses demonstrated, that PSI can improve the accuracy of component alignment and may reduce operative time and blood loss in comparison to surgery with standard instrumentation [5, 6]. However, the impact on implant longevity and revision rates remains to be determined.

Despite the debate about the optimal coronal alignment in TKA, achieving the planned alignment is considered an important determinant of a favourable outcome [7, 8]. In PSI‐TKA the three‐dimensional (3D) models and cutting jigs are based on CT or MRI scans performed with the patient in the supine nonweight bearing (NWB) position. In this position the loading forces across the knee joint are notoriously reduced significantly affecting the lower limb alignment [9, 10, 11, 12, 13].

León‐Muñoz et al. [14] investigated discrepancies between LLR and CT‐scan‐based 3D models of 227 knees prior to TKA and found only in 11% of the cases, that the hip–knee–ankle angle (HKA) matched between modalities. Another study compared LLR and NWB‐MRI of the whole leg and concluded, that the amount of articular wear and the load‐bearing axis seem to significantly influence the change of HKA between NWB and WB state [15].

In an attempt to address these differences between modalities, 2D/3D registration methods were developed [16, 17]. However, there is no commercially available and validated method on the market. In clinical practice, the surgeon needs to adapt the surgical correction based on 3D NWB models after considering the extent of preoperative deformity under WB conditions in LLR to achieve the “real” postoperative coronal alignment.

To assist the orthopaedic surgeon and in order to optimise preoperative planning in PSI‐TKA, the goal of this study is (1) to assess differences between 2D WB and 3D NWB planning in TKA and (2) to identify factors that influence intermodal differences.

## METHODS

### Study cohort

After ethical approval (2022‐02134), all consecutive patients that underwent surgery for a primary PSI‐TKA were screened between 2015 and 2020 (*n* = 332) in a single centre. Inclusion criteria comprised patients that underwent PSI‐TKA (Medacta SA, MyKnee GMK©) with preoperative X‐ray, CT, WB LLR and a complete preoperative planning protocol in 2D and 3D available. After excluding patients, without complete preoperative planning information (*n* = 5), missing demographic information (*n* = 2), intraoperative conversion to other TKA model (*n* = 2), 352 knees of 323 patients were available for final analysis.

Patients' medical records were reviewed and the following data were obtained: age at surgery, gender and body mass index (BMI). Preoperative range of motion was assessed and the passive extension deficit was graded into four different stages (1: <5°, 2: 5°−10°, 3: 10°−15°, 4: >15°).

### Radiographic analysis

#### 2D analysis

**Figure 1:**
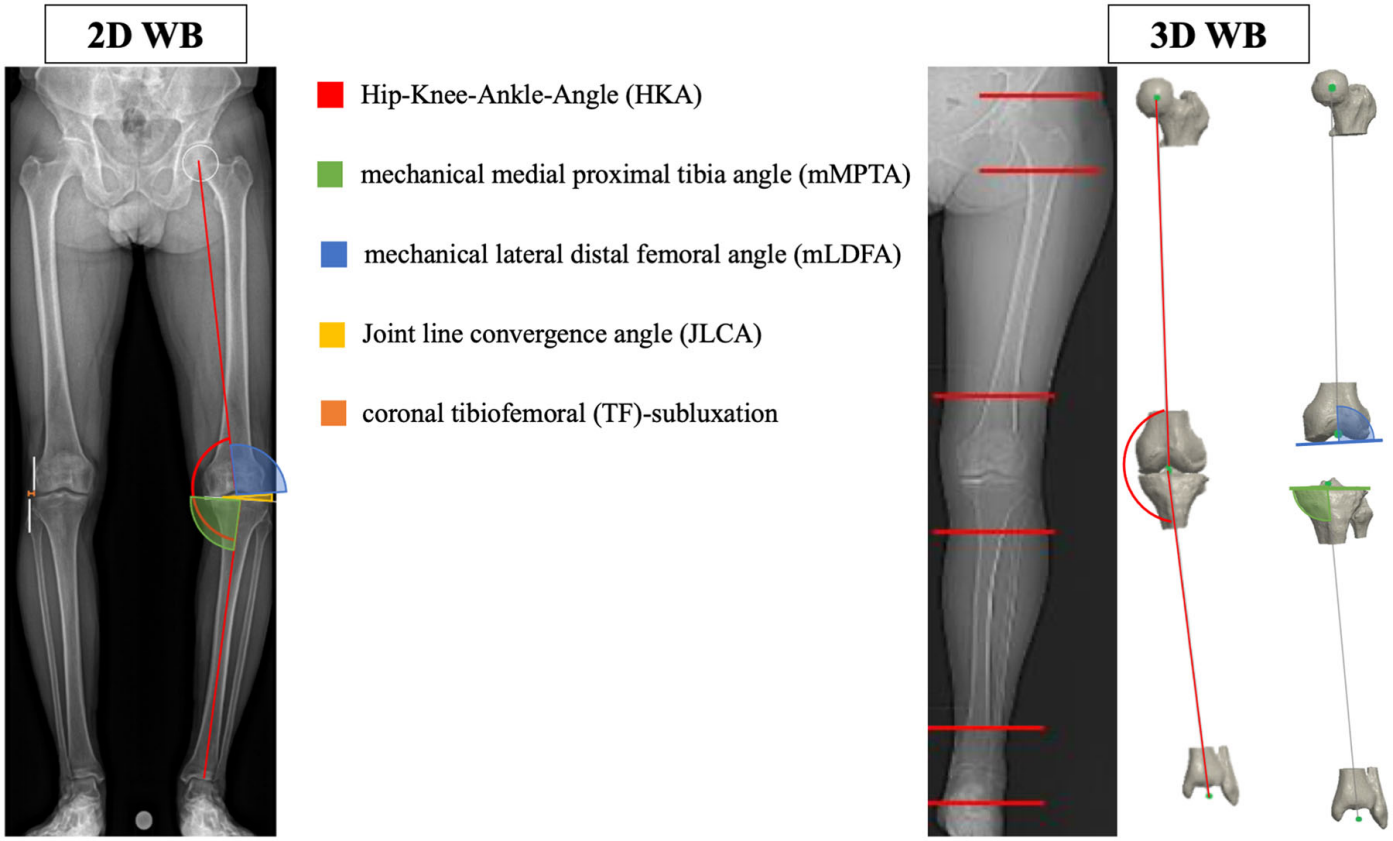
Illustration of radiographic assessment in 2D and 3D. Preoperative deformity analysis included hip–knee–ankle angle, mechanical medial proximal tibia angle, and mechanical lateral distal femoral angle. Joint line convergence angle as well as coronal tibiofemoral subluxation.

Preoperative planning of all patients was retrospectively reviewed and included routine radiographic assessment of WB LLR (EOS Imaging). The frontal LLR was imported to mediCAD® software (Hectec GmbH) and calibrated. After manually selecting the centre of the femoral head, apex of the greater trochanter, femoral and tibial joint line, medial and lateral border of the femoral condyles and tibial plateau, medial and lateral border of the talus, and the joint line of the talus, automatic deformity analysis is performed by the software according to Paley et al. [18]. Preoperative deformity analysis included HKA (°; + = varus, − = valgus), mechanical medial proximal tibia angle (mMPTA; °) and mechanical lateral distal femoral angle (mLDFA; °). Joint line convergence angle (JLCA; °; + = lateral opening, − = medial opening) as well as coronal tibiofemoral (TF)‐subluxation (mm) were measured in standing X‐ray (Figure 1) [19].

#### 3D analysis

All patients were scheduled for a PSI‐TKA using the MyKnee system of Medacta (Medacta SA, MyKnee GMK©). The preoperative planning is performed using a specific software based on preoperative CT in which only the hip, knee, and ankle joints were scanned (Figure 1). Slice thickness was 1.0 mm with an in‐plane resolution of 0.4 × 0.4 mm. CT scans were acquired with the patients in a supine position and the knee in full extension. Bony landmarks of the lower extremity are manually determined: hip centre, femur centre, medial and lateral epicondyles, medial and lateral distal condyles, medial and lateral posterior condyles and anterior cortex, tibia medial and lateral plateaus, tibia medial and lateral posterior wall, anterior/posterior plateaus points, tibia centre, ankle centre. By using a standardised protocol with defined landmarks, 3D alignment assessment is reliable with low intra‐ and interobserver variability [20]. Based on these bony landmarks, preoperative knee parameters are calculated: HKA, femoral mechanical axis, transepicondylar axis, femur external rotation and femur varus/valgus, posterior condyle axis, tibia mechanical axis, tibia varus/valgus, tibial slope. In accordance with the surgeon's preference bony resection parameters are simulated and possible implant sizes evaluated. Once the planning is finalised, patient specific cutting jigs are created for intraoperative use.

Femoral and tibial bone loss was graded according to Anderson in preoperative CT [21]. Type I are minor defects and contain cancellous bony defects. Type II defects are defined as moderate or severe cancellous and/or cortical bone defects, which can be further divided into Type IIA defects involving one femoral condyle or tibial plateau, while type IIB defects involve both femoral condyles and tibial plateaus. Type III lesions show massive cavitary and segmental bone loss of both tibial plateaus and/or femoral condyles with/without ligament or tendon involvement.

### Statistics

First, we sought to report the differences between the 2D WB and 3D NWB deformity analysis for TKA planning. The difference between 2D HKA and 3D HKA was classified into “correct” (difference between 2D HKA and 3D HKA < 1°), “underestimated” (difference between 2D HKA and 3D HKA > 1° and coronal deformity in 3D < 2D) and “overestimated” (difference between 2D HKA and 3D HKA > 1° and coronal deformity in 3D > 2D). Data are presented as mean ± standard deviation or median and interquartile range. RStudio (R version 3.6.2 (2019‐12‐12), PBC) was used for data processing and a *p* < 0.05 was considered statistically significant.

A quantile–quantile (Q–Q) plot was used to assess data normality.

Second, we analysed the influence of patient‐specific and radiographic factors based on the current “gold standard” of the 2D LLR (WB) on the reported absolute difference between 2D and 3D (NWB) HKA. A multivariable regression model was calculated using the difference between 2D/3D HKA as dependent variable. Backward stepwise selection was used to select the significant variables. The variables tested included age, gender, BMI, preoperative extension deficit, 2D HKA, mMPTA, mLDFA, JLCA, TF‐subluxation and femoral and tibial bone loss. The results are reported as odds ratios. Adjusted *R* ^2^ was calculated as a measure of the goodness of fit of the models.

As appropriate, the nonparametric Friedman test or the dependent *t*‐test was used to assess significant differences between two groups.

## RESULTS

Included patients had a mean age of 66.2 ± 9.7 years and 65.9% were female. The mean BMI was 31.4 ± 6.6 kg/m^2^ and a bilateral TKA was performed in 29 patients (8.2%).

### Differences between 2D/3D planning modalities

The coronal deformity differed significantly between 2D and 3D planning modalities (varus knees: *p* < 0.0001, valgus knees: *p* < 0.0001) (all radiographic parameters listed in Table 1).

**Table 1:** Difference between radiographic parameters in 2D and 3D for varus and valgus knees.

	Varus (*n* = 231)	Valgus (*n* = 121)
HKA (°)		
2D	9.9 ± 5.1	−8.2 ± 6.0
3D	6.7 ± 4.0	−5.5 ± 4.4
Mean absolute difference	3.4 ± 2.8	3.2 ± 3.8
	***p*** < **0.0001**	***p*** < **0.0001**
JLCA (°)		
2D	4.9 ± 2.7	−3.2 ± 3.9
3D	NA	NA
mMPTA (°)		
2D	84.3 ± 3.3	90.2 ± 3.4
3D	85.9 ± 3.1	89.7 ± 3.6
Mean absolute difference	2.4 ± 2.0	2.3 ± 2.3
	***p*** < **0.0001**	*p* = 0.06
mLFDA (°)		
2D	90.6 ± 2.3	84.6 ± 2.5
3D	88.5 ± 2.0	84.6 ± 2.1
Mean absolute difference	3.7 ± 2.6	1.2 ± 1.2
	***p*** < **0.0001**	*p* = 0.69
TF‐subluxation (mm)		
2D	5.9 ± 3.3	2.8 ± 3.4
3D	NA	NA

*Note*: Significant *p* values marked bold.

Abbreviations: 2D, two‐dimensional; HKA, hip–knee–ankle angle; JLCA, joint line convergence angle; mLFDA, mechanical lateral distal femoral angle; mMPTA, mechanical medial proximal tibia angle; NA, not applicable; TF, tibiofemoral.

**Figure 2:**
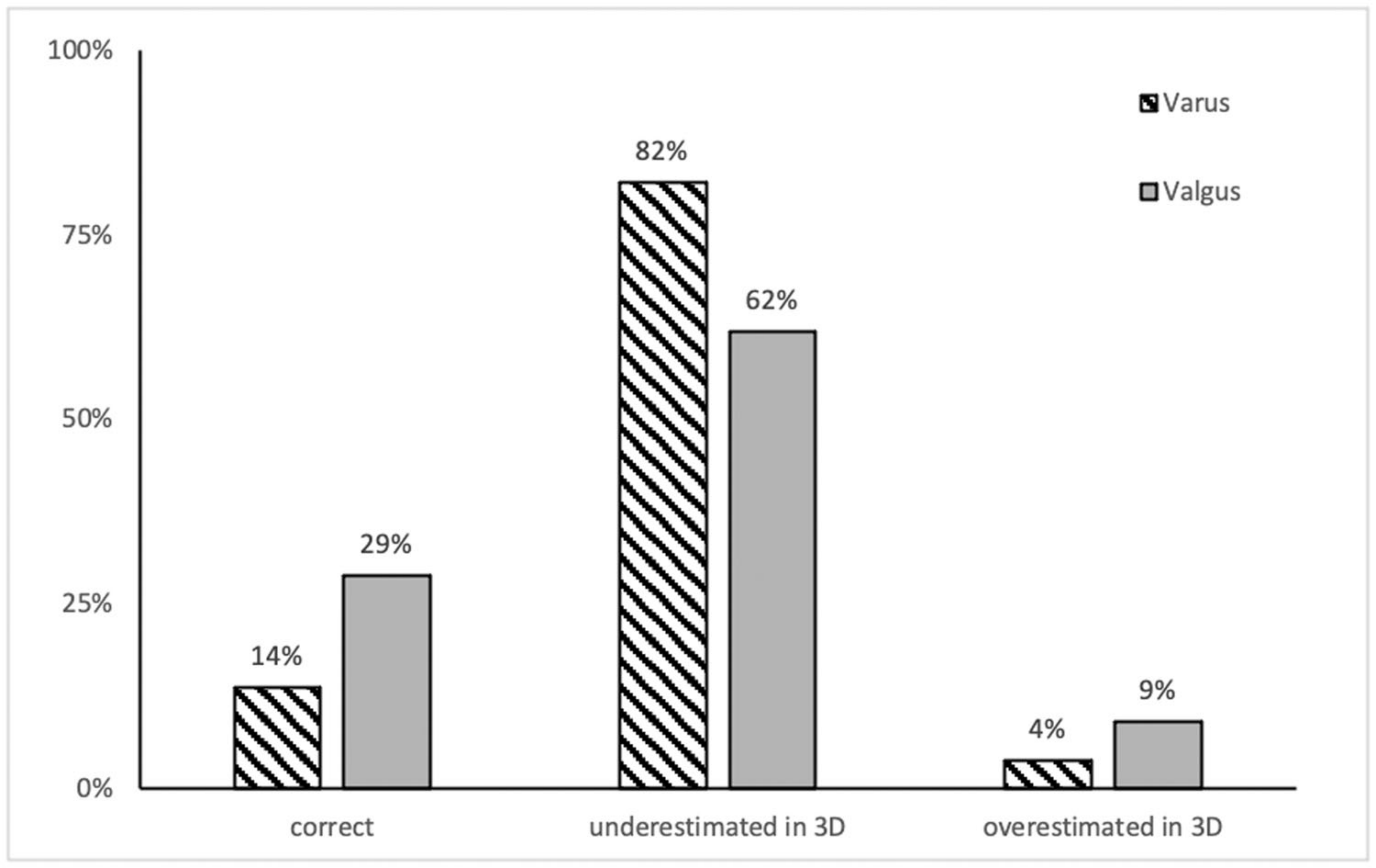
Difference between hip–knee–ankle angle in two‐dimensional (2D) and 3D for varus and valgus knees. Classified into “correct,” “underestimated” and “overestimated.”

In the majority of patients, the deformity was underestimated in 3D analysis (Figure 2). Only 14% of varus knees and 29% of valgus knees showed a difference between modalities less than 1° (Table 2).

**Table 2:** HKA‐difference between the two modalities classified in <1°, difference between 1° to 3°, >3° and 5° and >5°.

HKA‐difference between modalities	Varus (*n* = 231)	Valgus (*n* = 121)
<1°	14%	29%
1°−3°	39%	35%
>3°−5°	31%	16%
>5°	16%	20%

Abbreviation: HKA, hip–knee–ankle angle.

### Factors associated with the difference between 2D/3D planning modalities

**Figure 3:**
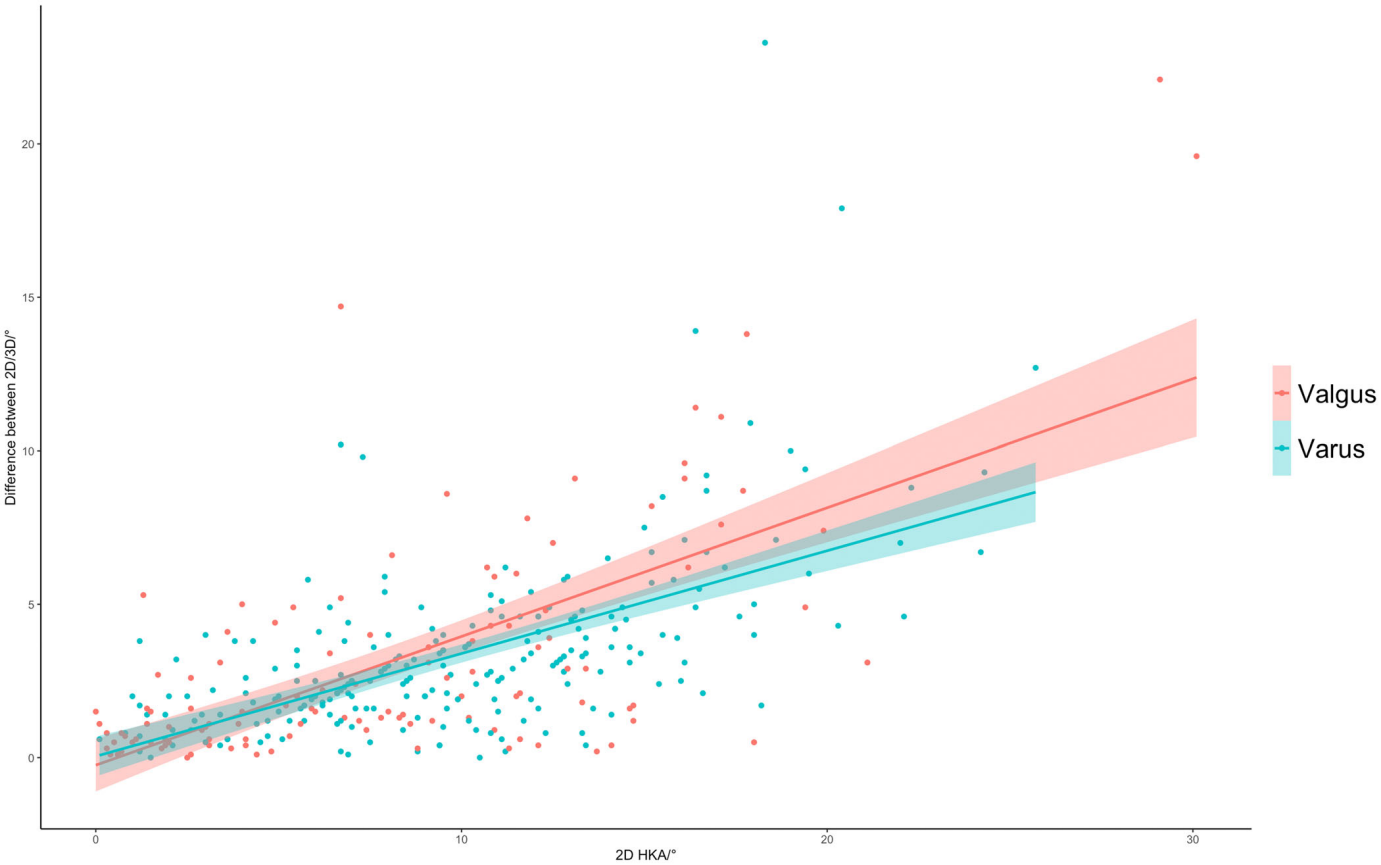
Scatterplot depicting the relationship between 2D/3D HKA (*y*‐axis) and 2D‐HKA (*x*‐axis) for valgus and varus knees. Increasing varus/valgus deformity is associated with increasing difference between 2D/3D HKA. 2D, two‐dimensional; HKA, hip–knee–ankle angle.

A higher valgus/varus deformity in 2D is associated with a significant increase of the difference between 2D/3D HKA (varus knees: *β* = 0.38; *p* < 0.0001 | valgus knees: *β* = −0.6; *p* < 0.0001) (Figure 3).

The JLCA is also associated with increasing difference between 2D/3D HKA (varus knees: *β* = 0.14; *p* = 0.03 | valgus knees: *β* = −0.3; *p* = 0.0001). All significant predictors of the multiple regression analysis are listed in Tables 3 and 4. The corrected goodness‐of‐fit of the regression models was moderate (varus knees: adjusted *R* ^2^ = 0.38|valgus knees: adjusted *R* ^2^ = 0.47).

**Table 3:** Multivariable regression analysis for **varus** knees with the difference between 2D/3D HKA as the dependent endpoint and after removing insignificant variables.

Varus (*n* = 231)		
Independent variable	Regression coefficient (*β*, 95% CI)	*p*‐Value
Patient demographics		
Age at surgery (years)	0.02 [−0.01; 0.05]	0.24
Gender (male)	0.42 [−0.21; 1.05]	0.19
Radiographic (2D)		
HKA (°)	0.38 [0.31; 0.45)	**<0.0001**
JLCA (°)	0.14 [0.01; 0.28]	**0.03**
Femorotibial subluxation (mm)	−0.06 [−0.16; 0.04]	0.25
Clinical		
Preop‐ROM	0.37 [−0.01; 0.76]	0.06

*Note*: Adjusted *R* ^2^ = 0.38, *p* < 0.0001. Significant *p* values marked in bold.

Abbreviations: 2D, two‐dimensional; CI, confidence interval; HKA, hip–knee‐ankle angle; JLCA, joint line convergence angle; Preop‐ROM, preoperative range of motion.

**Table 4:** Multivariable regression analysis for **valgus** knees with the difference between 2D/3D HKA as the dependent endpoint and after removing insignificant variables.

Valgus (*n* = 121)		
Independent variable	Regression coefficient (*β*, 95% CI)	*p*‐Value
Patient demographics		
BMI (kg/m^2^)	0.06 [−0.01; 0.13]	0.11
Radiographic (2D)		
HKA (°)	−0.60 [−0.76; −0.45]	**<0.0001**
JLCA (°)	−0.3 [−0.45; −0.15]	**0.0001**
Femorotibial subluxation (mm)	0.08 [−0.07; 0.22]	0.29
LDFA (°)	−0.28 [−0.54; −0.03]	**0.03**
mMPTA (°)	0.15 [−0.06; 0.35]	0.15

*Note*: Adjusted *R* ^2^ = 0.47, *p* < 0.0001. Significant *p* values marked bold.

Abbreviations: 2D, two‐dimensional; BMI, body mass index; CI, confidence interval; HKA, hip–knee‐ankle angle; JLCA, joint line convergence angle; LDFA, lateral distal femoral angle; mLFDA, mechanical lateral distal femoral angle; mMPTA, mechanical medial proximal tibia angle.

## DISCUSSION

The most important finding of this study is that the coronal alignment in preoperative 3D models for PSI‐TKA significantly differed from 2D WB state and the difference between modalities correlated with the extent of varus/valgus coronal deformity. In the vast majority of cases, the 3D NWB approach significantly underestimated the preoperative deformity, which needs to be considered to achieve the planned correction when using PSI in TKA.

León‐Muñoz et al. [14] also reported that CT scan‐based 3D models for PSI‐TKA underestimated the HKA in comparison to 2D WB LLRs in 74% of their cases. The extent of the mean absolute difference between modalities also showed a comparable distribution to the present findings. Further analysis regarding possible influencing factors on the difference between modalities were not conducted in the prior study.

Paternostre et al. [15] investigated differences between LLR and NWB MRI in 70 patients scheduled for PSI‐TKA. In contrast to León‐Muñoz et al. [14] and the present study, they only found a significant difference (>1°) between modalities in 46% of their patients. The influence of patient specific and radiographic parameters on the difference between modalities have been analysed separately. Patients with a higher Kellgren–Lawrence stage (3 or 4) showed a significant difference between 2D WB and 3D NWB HKA [15]. This is in line with our findings taking into account, that the JLCA is a surrogate representing the complex interlinking of surrounding soft tissue laxity and intra‐articular deformity arising from osteoarthritis [22, 23]. The advantage of using the JLCA is the (semi‐)automated software calculation, thus avoiding possible intra‐ and interobserver bias [24]. Paternostre et al. [15] reported also, that patients with a zone mechanical axis outside of the knee (Kennedy Zone 0 and 1 or Zone 4 and 5) differed significantly regarding 2D and 3D HKA. This can also be reconciled with our result, that the extent of varus/valgus deformity significantly influenced the mean absolute difference since the WB line correlates with HKA [25].

Despite the fact, that almost half of the varus knees and over one‐third of the valgus knees demonstrated a difference between modalities >3°, the postoperative occurrence of outliers (>3°) in CT‐based PSI‐TKA is significantly lower. Koch et al. [26] reported a total of 12.4% of outliers in the frontal plane in 291 cases. Other studies of CT‐based PSI‐TKA showed comparable low outlier rates [27, 28]. This might be due to the surgeon dependent adjustment to the PSI cutting jigs of the 3D models and/or intraoperative corrections. Apart from that, the extent of intra‐articular deformity as well as soft tissue laxity represented by JLCA, may not have such a decisive influence on postoperative alignment in TKA in contrast to joint‐preserving surgery.

Notwithstanding the intention to contribute to elucidate the differences between the two modalities, the calculated models could only explain less than 50% of the variance between 2D and 3D HKA. This might be due to the fact, that there is a large interindividual variability in osteoarthritic knees leading to the development of at least six varus and nine valgus phenotypes [29, 30]. The highly variable coronal tibial and femoral alignment might contribute to an unfavourable outcome, since these variations are not sufficiently considered in current total TKA alignment philosophies and preoperative planning [31]. Another reason for the differences between 2D and 3D HKA might be, that frontal leg alignment correlates with pelvic tilt and was not considered in the present study [32]. The influence of the position of the patient (supine vs. standing) will most probably differ in healthy compared to patients with hip or lower back pathology. Furthermore, in LLR axial limb rotation significantly influences the measured alignment especially in osteoarthritic knees with extension deficits [33]. For varus knees, the preoperative extension deficit remained after stepwise regression, but did not reach level of significance.

PSI‐TKA was introduced to significantly contribute to a more personalised and consistent alignment [4]. Meta‐analysis demonstrated, that 3D models improve the accuracy of rotational alignment in TKA and reduce the proportion of outliers from the target zone [5]. The big disadvantage about the acquisition of an additional dimension in knee surgery is, that it is usually not performed under “real” conditions, that is WB. Several studies demonstrated significant difference of lower limb alignment due to the position dependent loading forces across the knee joint [9, 10, 11, 12, 13]. In order to provide a solution for this problem, 2D/3D registration methods were developed to combine the different modalities [16, 17]. Based on this technology, Roth et al. [34] developed an algorithm to transform NWB 3D models of lower limbs to a WB state. This might be the future for PSI‐TKA to be able to use the advantages of 3D imaging under real conditions, omitting common causes of revision for TKA and consequently leading to a superior patient outcome. However, in clinical practice neither the hardware nor the software are commonly available.

The results of the present study can therefore contribute to optimising preoperative planning in PSI‐TKA and to achieve the planned alignment. On the basis of a commonly available, standardised and reliable deformity analysis in a large patient cohort, the study demonstrated that the coronal alignment of preoperative 3D models significantly differs from 2D assessment. The difference between modalities correlates with the extent of frontal deformity as well as the JLCA. The influence of WB state should not be underestimated and an LLR performed for preoperative planning in PSI‐TKA.

Limitations of the study are its retrospective design. The preoperative planning was performed using a specific software by one manufacturer and based on manually determination of bony landmarks in preoperative NWB CT. By using standardised definitions for each landmark the reliability to obtain reproducible alignment parameters is high, but cannot simply be transferred to all 3D models [20]. Preoperative passive extension deficit was considered as clinical parameter, since extension deficits of the knee can significantly influence the coronal lower limb alignment especially in combination with (mal‐)rotation [33, 35, 36]. The differences between planned and achieved (postoperative) coronal correction as well as the influence of the surgeon dependent adjustment was not part of this study.

## CONCLUSION

The coronal alignment in preoperative 3D model for PSI‐TKA significantly differed from 2D WB state and the difference between modalities correlated with the extent of varus/valgus deformity. In the vast majority of cases, the 3D NWB approach significantly underestimated the preoperative deformity, which needs to be considered to achieve the planned correction when using PSI in TKA.

## AUTHOR CONTRIBUTIONS

Patrick Pflüger carried out the data curation, statistical analysis and drafted the manuscript. Sandro Hodel participated in the design of the study and helped to draft the manuscript. Stefan M. Zimmermann and Svenja Knechtle helped with data acquisition and processing. Lazaros Vlachopoulos and Sandro F. Fucentese conceived of the study, and participated in its design and coordination. All authors read and approved the final manuscript.

## CONFLICT OF INTEREST STATEMENT

Sandro F. Fucentese is a consultant for Medacta SA (Switzerland), Smith & Nephew (United Kingdom), Zimmer Biomet and Karl Storz SE & Co. KG (Germany). The other authors have no conflict of interest to declare.

## ETHICS STATEMENT

The study was conducted in accordance with the Declaration of Helsinki and approved by the local Ethics Committee (2022‐02134). Written informed consent was obtained from all patients.
